# MLL-SEPT5 Fusion Transcript in Myelodysplastic Syndrome Patient With t(11;22)(q23;q11)

**DOI:** 10.3389/fmed.2021.783229

**Published:** 2021-12-22

**Authors:** Duobing Zou, Ying Chen, Ningning Wu, Yi Zhang, Guifang Ouyang, Qitian Mu

**Affiliations:** ^1^Laboratory of Stem Cell Transplantation, Ningbo First Hospital, Ningbo, China; ^2^Department of Hematology, Ningbo First Hospital, Ningbo, China

**Keywords:** myelodysplastic syndrome, MLL rearrangement, septin, SEPT5, t(11;22)(q23;q11)

## Abstract

**Objectives:** This study aimed to identify unknown mixed lineage leukemia (MLL) translocation partner genes in a *de novo* patient with myelodysplastic syndrome (MDS) with t(11;22)(q23;q11) and investigate the clinical and molecular features of this patient.

**Methods:** Bone marrow cells were assessed by karyotype analysis to reveal chromosomal abnormalities. Fluorescence *in situ* hybridization (FISH) was performed to detect MLL gene rearrangement using an MLL-specific break-apart probe. LDI-PCR and RT-PCR were performed, and the PCR products were sequenced using an Illumina MiSeq sequencer (Illumina, San Diego, CA, USA). The sequence data of the PCR products were analyzed using bioinformatics tools. Meanwhile, clinical data were collected to evaluate the prognosis of the patient.

**Results:** Chromosomal karyotype analysis showed that the karyotype of the patient was 46, XX, t(11;22)(q23;q11)[10]/46, XX[1]. Subsequently, FISH data confirmed MLL gene rearrangement in the patient. LDI-PCR precisely showed that SEPT5 was the MLL translocation partner gene. RT-PCR and sequencing analysis disclosed the presence of MLL-SEPT5 fusion transcript and confirmed the fusion between MLL exon 8 and SEPT5 exon 3. Moreover, the patient had a recurrence shortly after allogeneic hematopoietic stem cell transplantation.

**Conclusion:** Although the MLL-SEPT5 fusion transcript was occasionally described in acute myeloid leukemia, it was first identified in MDS. Patients with MLL-SEPT5 fusion gene exhibited a poor prognosis even with an aggressive treatment.

## Introduction

Myelodysplastic syndromes (MDS) are clonal myeloid malignancies characterized by ineffective hematopoiesis, refractory cytopenia, and an increased risk of progression to acute leukemia (1). Chromosomal translocations involving the mixed lineage leukemia (MLL) gene are the most frequent genetic alteration in adult and pediatric leukemia (2), especially in pediatric leukemia, which is reported to have an incident rate of up to 70% (3). In contrast to acute leukemia, MLL rearrangement occurs rarely in patients with MDS. Multiple studies have demonstrated that most patients with acute leukemia with MLL rearrangements are characterized by a high degree of malignancy, low rates of remission, insensitive to chemotherapy, and poor prognosis. Accordingly, unambiguous identification of MLL rearrangements is useful not only for diagnosis at an early stage but also for prognostic evaluation.

Balanced chromosomal translocations are the most common rearrangements for MLL. Because of the promiscuous features of MLL, the fusion partner genes diversity was determined. MLL fusion partner genes are divided into 4 classes: nuclear proteins (such as AF9, AF10, and ENL), cytoplasmic proteins (such as EPS15, SH3GL1, and GAS7), histone acetyltransferases (such as EP300 and CREBBP), and septin gene family members (such as SEPT5, SEPT6, and SEPT9). So far, over 100 different MLL reciprocal translocations have been described and approximately 80 partner genes for MLL have been identified at the molecular level (4). Among them, AF4, AF9, and ENL were the most frequent fusion partner genes with MLL. However, many uncommon MLL rearrangements are easily overlooked during the diagnostic encounter. Our study elucidated that the MLL gene at band 11q23 was involved in chromosome translocations with the 22q11-22q13 region of chromosome 22. Existing literature has found that SEPT5 or EP300 is located at 22q11-22q13 and acts as the translocation partner of MLL (5, 6). As expected, SEPT5 has been precisely identified as a partner of the MLL gene in our patient using LDI-PCR. Patients with MLL gene rearrangement are classified as a distinct subcategory in acute leukemia according to the classification of 2008 WHO (7). However, patients with MDS with *MLL* gene rearrangement, especially MLL-SEPT fusion, have not been well defined.

Here, we reported a case of MDS with t(11;22)(q23;q11) abnormal karyotype in a 46-year-old woman and confirmed the presence of an MLL-SEPT5 fusion transcript. To our knowledge, the MLL-SEPT5 fusion transcript has not yet been reported in MDS.

## Patient and Methods

### Patient

In June 2020, a 46-year-old, previously healthy woman was evaluated at a local hospital for leukopenia. After symptomatic support treatment for 1 month, no improvement in leukocyte numbers was observed, then the patient was admitted to our hospital (Ningbo First Hospital, Ningbo, China) for further examinations. She denied any symptom of fever, respiratory disorder or other symptoms such as skin rashes and joint pain before the onset. Moreover, she did not have any history of exposure to ionizing radiation, chemotherapy or toxic substances, and again no history of malignancy, alcohol, tobacco, or drug addiction. A complete blood count was carried out using routine automated analyzers and showed a decrease in the erythroid and myeloid lineage (hemoglobin of 9.7 g/dl, white blood cell count of 1.65 × 10^9^/L, absolute neutrophil count of 0.4 × 10^9^/L, and a platelet count of 132 × 10^9^/L) in the peripheral blood. Bone marrow smears showed granulocytic-lineage dysplasia, containing 1% myeloblast, 15% monoblasts, and promonocytes. Flow cytometry data showed that the blasts were positive for CD45, CD15, CD13, CD33, and CD38 and negative for lymphoid markers. A diagnosis of MDS-EB was established according to classification of the 2016 WHO. The patient with MDS was classified as very high IPSS-R risk (score: 6.5) and high WPSS risk (score: 4) at the time of diagnosis.

After a detailed assessment by hematologists, she received allogenic hematopoietic stem cell transplantation (allo-HSCT) on August 26, 2020. Before transplantation, the patient received myeloablative conditioning regimens (azacitidine plus modified busulfan/cyclophosphamide plus ATG). The patient underwent allo-HSCT from unrelated cord blood, peripheral blood stem cells and bone marrow (HLA6/10) of her daughter, followed by cyclosporine A, mycophenolate mofetil, and short-term methotrexate for prophylaxis of graft vs. host disease (GVHD). The patient received supportive care, including red blood cell transfusions, platelet transfusions, and treatment with recombinant human granulocyte colony stimulating factor (rhG-CSF) and thrombopoietin (TPO). In March 2021, the patient presented with chills, sputum expectoration, and cough under no obvious inducement. In total, 1 month later, the patient successively developed fever (38°C), pulmonary fungal infections, and extensive skin chronic GVHD. Thus, cefoperazone combined micafungin was prescribed. Unfortunately, she experienced a relapse 10 months after transplantation. The patient received 100 mg of azacitidine on day 1–7, and was subsequently treated with a PD-1 inhibitor combined with peripheral blood stem cells. In October 2021, the disease progressed to AML, and the evaluation of bone marrow showed 27% of blasts. The patient subsequently received decitabine in combination with cytarabine.

### Cytogenetic Analysis

The moderate numbers of BM cells were cultured in a commercial cell culture medium (Tianjin Reagent Biotech Corporation Ltd., Tianjin, China) at 37°C for 24 h. The cells were processed with colchicine, centrifuged, and treated with hypotonic solution (0.075 M KCl) at 37°C for 40 min. Then, the cell suspension was prefixed and finally fixed using methanol and acetic acid. Finally, the metaphase chromosomes were R-banded, stained with Giemsa, and scanned on a fully automated GSL-120 Leica microscope (Leica, Germany) for karyotype analysis. The chromosomes were described according to the International System for Human Cytogenetics Nomenclature (ISCN 2016).

### Fluorescence *in situ* Hybridization

After centrifugation, BM cells were harvested and resuspended in hypotonic KCl solution at 37°C for 40 min. The cells were pre-fixed, fixed, and stored at 4°C until the fluorescence *in situ* hybridization (FISH). FISH was performed on blasts from the BM samples according to product instructions using the MLL break-apart probe (Guangzhou LBP, China). Separation of the green and red fluorescence signals indicates the chromosomal rearrangements in MLL/11q23. At the same time, BCR/ABL fusion analysis was conducted as a control using BCR/ABL dual-color, dual-fusion probe (Guangzhou LBP, China).

### Long Distance Inverse-Polymerase Chain Reaction Analysis

Deoxyribonucleic acid was extracted from cell suspensions that were used for karyotype analysis. DNA samples were digested by the restriction enzyme Bam HI (Takara, Japan). Subsequently, phenol-chloroform was used to remove the residual Bam HI enzymatic activity. After purification, the digested linear DNA was religated into a circular DNA molecule at 16°C overnight using T4 DNA ligase (Takara, Japan). The religated DNA samples were terminated for 10 min at 65°C, then stored at −80°C until the LDI-PCR. PCR amplifications were conducted with TaKaRa LA Taq DNA Polymerase Kit (Takara, Japan) and following the instructions of the manufacturer. MLL gene-specific oligonucleotides sequences (Supplementary Table 1) were obtained from Prof Rolf Marschalek, and were used according to their experimental method in a previously published article (8). In total, 5 μl of religated DNA samples were used for LDI-PCR in a total volume of 50 with 5 μl of LA Taq Buffer, 5 μl of MgCl_2_ (25 mM), 8 μl of dNTP Mixture (2.5 mM each), 0.5 μl of TaKaRa LA Taq, 2 μl of forward primer, 2 μl of reverse primer, and 22.5 μl of RNase-free H_2_O. PCR amplification was carried out at 94°C for 1 min, followed by 30 cycles at 98°C for 10 s, 68°C for 10 min, 72°C for 10 min using the Bio-Rad PCR instrument (Bio-Rad, CA, USA). The LDI-PCR products were electrophoresed on a 0.8% agarose gel, and were then sequenced by Shanghai Sangong Biotech Corporation (Shanghai, China). The sequencing results were performed by alignment with BLAST searches of NCBI databases to determine the fusion site of MLL and MLL partner gene.

### RT-PCR and Sequencing of PCR Product

Blast cells were isolated from the samples of BM using Ficoll-Paque Plus (GE Healthcare, Sweden). RNA was extracted using RNAiso Plus reagent (Takara, Japan), and reverse transcription was performed with a cDNA Synthesis Kit (Thermo Scientific, Waltham, MA, USA) according to the instructions. Primers were synthesized by Tsingke Biotechnology (Hangzhou, China). Sequences of the primers are as follows: MLL-E7, 5′-TACAGGACCGCCAAGAA-3′ from exon 7 of MLL; SEPT5-E3, 5′-CAAAGCCTTTCTTCACCGAC-3′ from exon 3 of SEPT5; SEPT5-E5, 5′-TGTCCACGATGGTGAGCTTC-3′ from exon 5 of SEPT5. About 1 μl cDNA was used for amplification in a total volume of 20 with 0.8 μl of forward primer, 0.8 μl of reverse primer, 10 μl of SYBR Green (Takara, Japan), 0.4 μl of ROX, and 7 μl of RNase-free H_2_O. PCR procedures were carried out as described previously (9). The products were analyzed by electrophoresis with a 1% agarose gel and were then sequenced by the Shanghai Sangong Biotech Corporation (Shanghai, China). The product sequencing results were analyzed using Splign and BLAST searches of NCBI databases.

## Results

**Figure 1:**
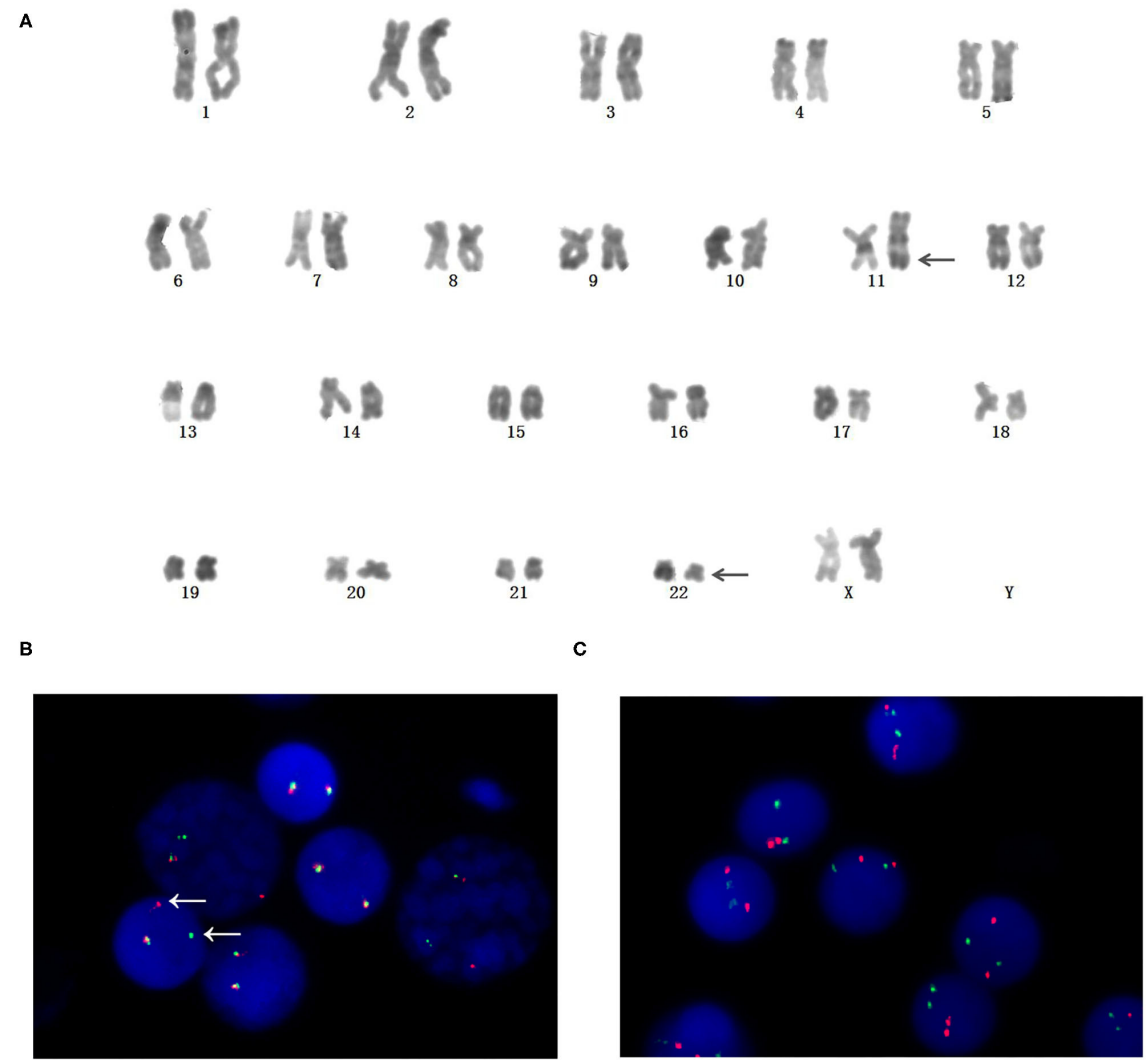
Identifification of chromosome rearrangements involving mixed lineage leukemia (MLL)/11q23. **(A)** Karyotype (R-banded) on bone marrow cells. Arrowheads indicate der(11) and der(22). **(B)** MLL rearrangement was confifirmed by Fluorescence *in situ* hybridization (FISH). Dissociation of a green and a red signal indicated the rearrangement of MLL. **(C)** BCR rearrangement was negative by FISH analysis.

Cytogenetic analysis by R-banding stain showed an abnormal karyotype: 46, XX, t(11;22)(q23;q11)[10]/46, XX[1]. The result is shown in Figure 1A. FISH analysis was performed to validate the existence of MLL rearrangements. The break-apart probe to 11q23 shows 1 normal fusion signal and dissociation of the 5′ MLL green and 3′ MLL red components of the other signal (arrows), suggesting that a translocation had occurred involving the MLL gene (Figure 1B). In order to exclude BCR (chromosome region 22q11) variants, FISH analysis for BCR-ABL was performed using dual-fusion probe, and BCR rearrangement was negative (Figure 1C).

**Figure 2:**
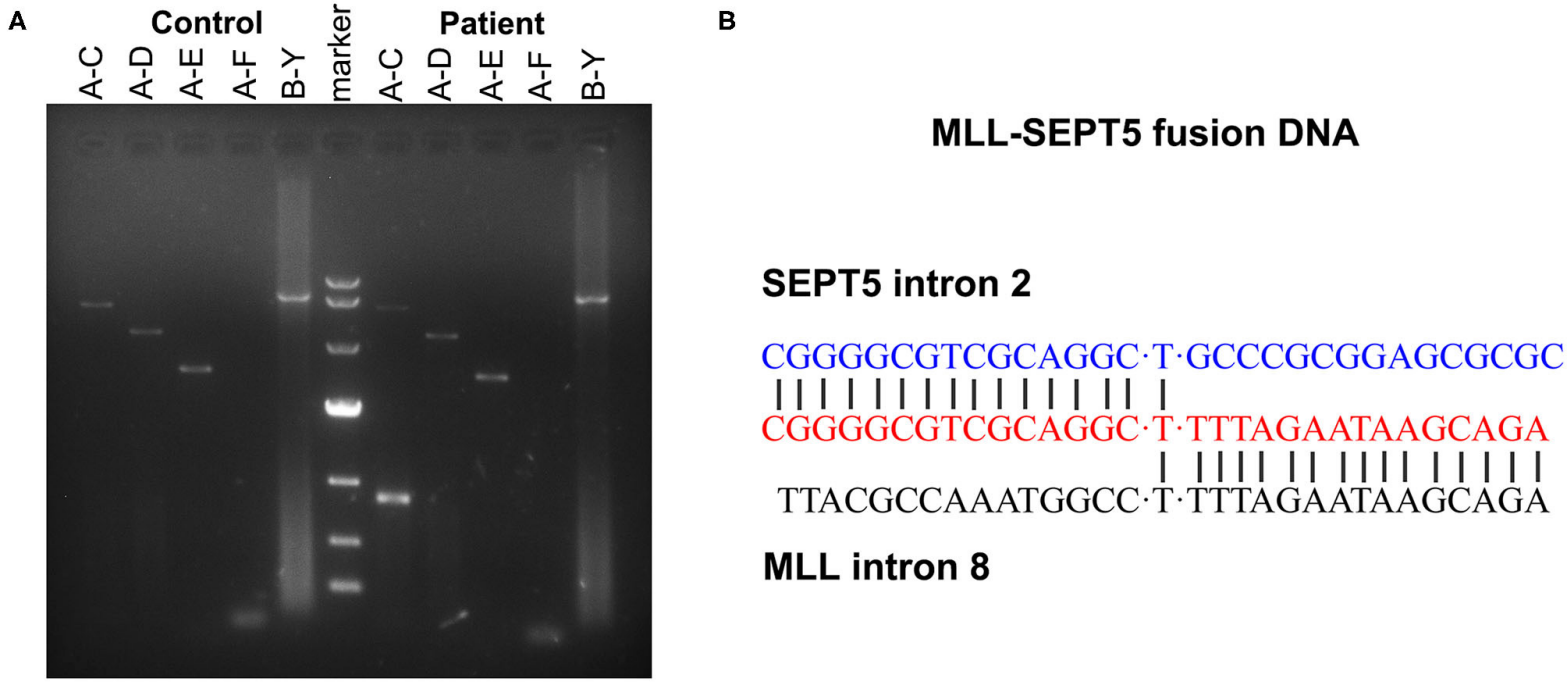
LDI-PCR analyses of MLL translocation partner gene. **(A)** Genomic DNA of healthy individual and the patient were tested with five different primer combinations (A–C, A–D, A–E, A–F and B–Y). (Left) control DNA (healthy individual peripheral blood). (Middle) DNA marker (TaKaRa, 3584A). (Right) the patient. **(B)** Sequence analysis of the SEPT5-MLL fusion gene showing juxtaposition of SEPT5 intron 2 and MLL intron 8. “T”was used to connect the two broken chromosomes.

**Figure 3:**
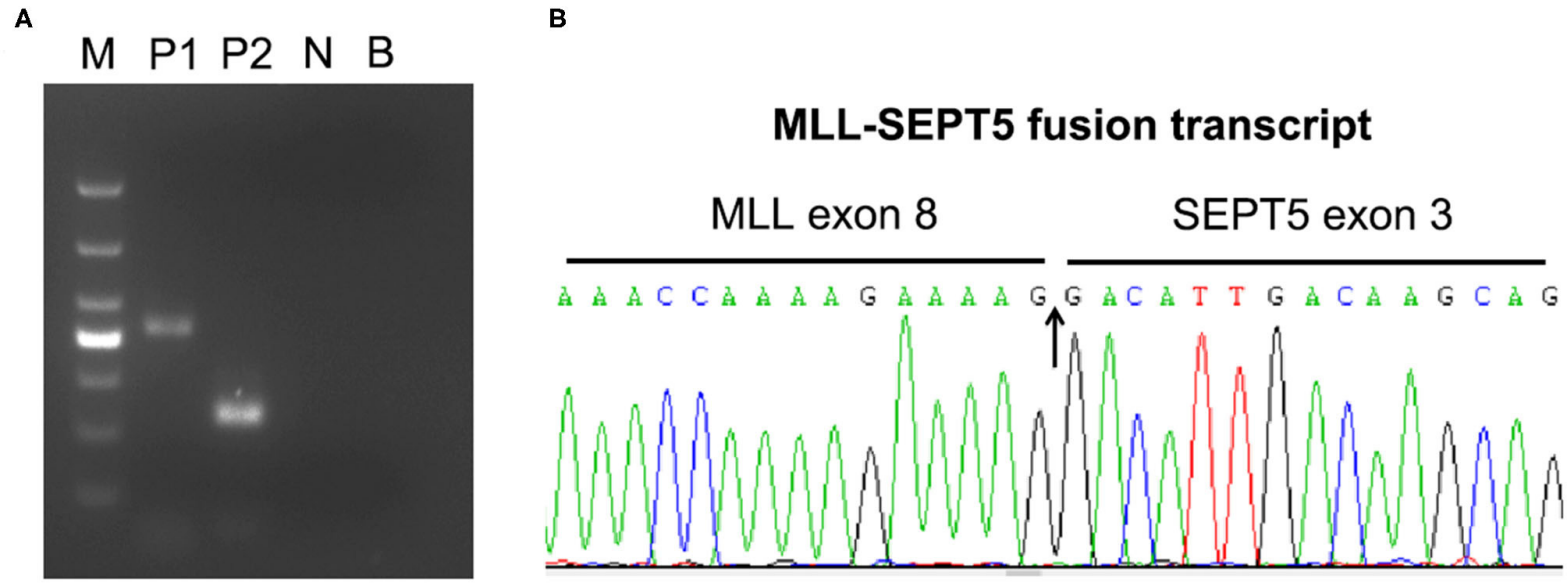
RT-PCR analyses of MLL-SEPT5 fusion transcript. **(A)** The products were detected with 2% agarose gel electrogenesis after amplification. M, DNA marker (TaKaRa, 3591A); P1, patient sample (primer combination: MLL-E7 and SEPT5-E5); P2, patient sample (primer combination: MLL-E7 and SEPT5-E3); N, PCR control; B, blank control. **(B)** Sequence analysis of the SEPT5-MLL fusion transcript showing juxtaposition of MLL exon 8 and SEPT5 exon 3.

To identify the involved partner gene in the current patients with MDS, LDI-PCR was performed using MLL gene-specific oligonucleotides. The amplification PCR product was separated by horizontal electrophoresis and obtained an 815 bp product in length (Figure 2A). Sequencing analysis indicated that intron 2 of SEPT5 is fused to intron 8 of MLL on the DNA molecule (Figure 2B). Furthermore, RT-PCR and direct sequencing were performed using MLL and SEPT5 specific primers to detect MLL-SEPT5 fusion transcript in the BM cells. As expected, sequencing and bioinformatics analysis revealed that exon 8 of MLL is fused to exon 3 of SEPT5 (Figure 3B), and a 416 bp and 215 bp product was observed when amplified using 2 different sets of primers, respectively (Figure 3A).

To better understand the possible roles of leukemogenesis of MLL-SEPT5 fusion, the main clinical and molecular features of MLL-SEPT5 fusion-positive patients are summarized in Table 1. Of the 10 MLL-SEPT5 fusion patients, 3 were infants, and seven were adults. The blast cells of these patients in the bone marrow showed myeloid phenotype. Impressively, our patient was diagnosed with MDS, while others were all diagnosed with AML. Furthermore, the prognosis for these patients with MLL-SEPT5 fusion seems poor, even if some of them had undergone stem cell transplantation.

**Table 1:** Clinical and molecular features of patients with MLL-SEPT5.

**Patient**	**Age/sex**	**Diagnosis**	**Immunophenotype**	**MLL-SEPT5 fusion transcript**	**Prognosis**	**References**
1	11.5months/F	AML	Not recorded	MLL exon 7-SEPT5 exon 3	Not recorded	Megonigal et al. (5)
2	13months/F	AML	Not recorded	MLL exon 7-SEPT5 exon 3	Not recorded	Megonigal et al. (5)
3	39years/M	AML	Not recorded	MLL exon 6-SEPT5 exon 4	OS: 12 months	Tatsumi et al. (14)
4	44years/F	AML	CD45(dim)/HLA-DR/CD38/CD15/CD33/CD13/CD11b/CD64/CD4	MLL exon 8-SEPT5 exon 3	CR by induction chemotherapy	Wang et al. (15)
5	32years/M	AML	CD33/CD64/CD117/HLA-DR/CD15/MPO/CD13	MLL exon 9-SEPT5 exon 3	OS: 7 months	Gao et al. (16)
6	32months/F	AML	CD45/MPO/CD15/CD33/CD116/HLA-DR	MLL exon 10-SEPT5 exon 3	Survived >2 years after transplantation	Launay et al. (17)
7	21years/M	AML	CD45/CD33/CD117/CD15/HLA-DR	MLL exon 8-SEPT5 intron 2	Transplantation, in complete remission	Wang et al. (18)
8	22years/F	AML	CD45/CD14/CD13/CD33	MLL exon 10-SEPT5 exon 3	Not recorded	Wang et al. (18)
9	43years/F	AML	CD4/CD11b/CD15/CD33/HLA-DR/	MLL-SEPT5(FISH)	OS: 2 months	Elzamly et al. (19)
10	46years/F	MDS	CD45/CD15/CD13/CD33/CD38	MLL exon 8-SEPT5 exon 3	Relapsed 10 months after transplantation, progressed to AML	Present

## Discussion

Many common fusion gene detections, including MLL fusion, have already been applied as routine examinations for the patients with hematologic malignancies in clinical practice. However, some rare fusion genes are still challenging to be found in patients. Here, we have reported a patient with MDS with t(11;22)(q23;q11), which resulted in an MLL-SEPT5 fusion.

Septins are GTP-binding proteins associated with crucial biological processes such as cytokinesis, membrane dynamics, and cytoskeletal reorganization (10). Increasing reports link septins to cancer in humans, including hematological malignancies (11). However, the exact function of septins in hematologic tumors remains incompletely known. In total, 5 septin genes (SEPT2, SEPT5, SEPT6, SEPT9, and SEPT11) are involved in chromosome translocations with MLL in patients with hematological malignancies, producing oncogenic MLL-fusion protein (12). As the MLL fusion partner, SEPT5 has been reported to be aberrantly expressed in acute myeloid leukemia (AML) (13). MLL-SEPT5 fusion transcript was the first reported in AML of infant twins with t(11;22)(q23;q11.2) (5). RT-PCR and subsequent products sequencing demonstrated that exon 7 of MLL is fused to exon 3 of SEPT5. Previous research has also indicated that the breakpoints in the MLL gene always occurs in the 6–11 exons (12), and as a partner gene of MLL, the SEPT5 gene easier occurs in the 5′ region (exons 1–3), resulting in the fusion that contains the entire open reading frame of the SEPT5 gene. Tatsumi et al. (14) reported that the SEPT5 gene fused to MLL in an adult patient with *de novo* AML and also found that SEPT5 expression in AML was markedly higher than that in ALL cell lines. Almost a decade later, the fourth patient with MLL-SEPT5 fusion transcript was reported by Wang et al. (15). We believe that some cases with MLL-SEPT5 fusion are easily ignored during that time due to its rarity. With more attention in the clinic, a growing amount of MLL-SEPT5 fusion has been discovered and identified in the recent years (16–19). The breakpoint of the MLL gene in 2 cases was same as that in our patient, while the breakpoint of the SEPT5 gene in 6 cases. Although the splice of the MLL-SEPT5 fusion gene in different ways in these patients, it is worth noting that involvement of SEPT5 exon 3 occurred with high frequency.

In addition, a high proportion (16%) of marrow blast cells was observed in our case. It is unclear whether our patient represented evolving AML or whether these patients with AML with MLL-SEPT5 fusion had a short history of MDS. In some unique entities (such as AML1-ETO, PML-RARa, and CBFβ-MYH11), the fusion gene is considered to be AML-defining according to the classification of 2008 WHO (7). Indeed, numerous clinical studies showed that almost all MLL-SEPT fusion genes had been identified in AML or therapy-related AML (20–22). Only 1 article reported that the identified fusion MLL-SEPT9 may be associated with *de novo* MDS until now. Interestingly, the authors also considered that the patient with MDS actually represented evolving AML that was discovered earlier than usual (23). For this reason, our patient was sufficient for the diagnosis of AML regardless of the bone marrow or peripheral blood blast cell proportion. If our view was true, it is time to classify MLL-SEPT, at least MLL-SEPT5, as a new provisional entity in AML.

Mixed lineage leukemia rearrangements are considered as an adverse risk factor and are often associated with adverse prognosis in acute leukemia (24). Currently, the effect of MLL rearrangements on the MDS prognosis remains poorly understood. In our study, the patient received an allogeneic hematopoietic stem cell transplantation. Unfortunately, she suffered a relapse of the disease after 10 months of transplantation. Most significantly, we found that MLL-SEPT5 fusion transcript is abundantly expressed in the bone marrow blast cells of the patient by RT-PCR. The patient eventually progresses to AML, as expected. Thus, MLL rearrangements involving the SEPT5 gene appear to be associated with a high risk for AML progression in MDS and an increased incidence of relapse. Because per leukemic cell carry only 1 copy of MLL-SEPT5 fusion on the DNA molecule, it is also suggested that detection of specific fusion sequences of patients may be helpful to monitor minimal residual disease and thus to take measures in time to prevent relapse.

In conclusion, although evidence from early studies (21, 25) suggested that MLL-SEPT fusion formation was likely due to this hypothesis that exposure to drugs targeting topoisomerase II can result in double-strand DNA breaks that trigger the error-prone non-homologous end joining pathway, the molecular mechanism that whether MLL-SEPT5 fusion can induce the transformation of MDS to AML require further investigations through experiments.

## Data Availability Statement

The original contributions presented in the study are included in the article/supplementary materials, further inquiries can be directed to the corresponding author.

## Ethics Statement

The studies involving human participants were reviewed and approved by the Medical Ethics Committee of Ningbo First Hospital. The patients/participants provided their written informed consent to participate in this study. Written informed consent was obtained from the individual(s) for the publication of any potentially identififiable images or data included in this article.

## Author Contributions

DZ performed most of the experiments. Experimental analysis was performed by YC and YZ. QM and GO conceived and designed the study. NW edited the manuscript. All authors contributed to the study and approved the final manuscript.

## Funding

This work was supported by Zhejiang Provincial Natural Science Foundation of China (No. LY20H080001), the Medical and Health Science and Technology Projects of Zhejiang Province (Nos. 2019KY170 and 2021KY997), and the Natural Science Foundation of Ningbo (No. 2016A610152).

## Conflict of Interest

The authors declare that the research was conducted in the absence of any commercial or financial relationships that could be construed as a potential conflict of interest.

## Publisher's Note

All claims expressed in this article are solely those of the authors and do not necessarily represent those of their affiliated organizations, or those of the publisher, the editors and the reviewers. Any product that may be evaluated in this article, or claim that may be made by its manufacturer, is not guaranteed or endorsed by the publisher.
