# Supplementary material for: MLL-SEPT5 Fusion Transcript in Myelodysplastic Syndrome Patient With t(11;22)(q23;q11)
Source: Front Med (Lausanne). 2021 Dec 22;8:783229. doi: 10.3389/fmed.2021.783229 (PMC8729882; doi:10.3389/fmed.2021.783229)
Supplement: Supplementary file 1 [file Table_1.DOCX]

Supplementary Material

# Supplementary Tables

**Supplementary Table 1.** Primer sequences for der(11) LDI-PCR

| Primer | Primer sequences (5'to3') |
| --- | --- |
| A | 5`-gacattcccttcttcactcttttcctc-3` |
| B | 5`-gcagcctccaccaccagaatcaggtgagtg-3` |
| C | 5`-cccacatgttctagcctaggaatctgc-3` |
| D | 5`-atcctgaataaatgggacctttctgttggtgg-3` |
| E | 5`-ctcttttccgtcttaatacagtgctttgcacc-3` |
| F | 5`-ttgtgagcccttccacaagttttgtttagagg -3` |
| Y | 5`-gtcccaggcactcagggtgatagctgtttcgg-3` |

PCR reactions and fragment sizes

AC: 6.6 kb

AD: 4.7 kb

AE: 3.1 kb

AF: 1.6 kb

control reaction = BY: 7.9 kb
